# Visual Motion Coherence Responses in Human Visual Cortex

**DOI:** 10.3389/fnins.2022.719250

**Published:** 2022-03-02

**Authors:** Andriani Rina, Amalia Papanikolaou, Xiaopeng Zong, Dorina T. Papageorgiou, Georgios A. Keliris, Stelios M. Smirnakis

**Affiliations:** ^1^Department of Neurology Brigham and Women’s Hospital and Jamaica Plain Veterans Administration Hospital, Harvard Medical School, Boston, MA, United States; ^2^Visual and Cognitive Neuroscience, Faculty of Science, University of Tübingen, Tuebingen, Germany; ^3^Department of Experimental Psychology, Institute of Behavioral Neuroscience, University College London, London, United Kingdom; ^4^Department of Neuroscience, Baylor College of Medicine, Houston, TX, United States; ^5^Department of Physical Medicine and Rehabilitation, Neuroscience, Psychiatry Baylor College of Medicine, Houston, TX, United States; ^6^Department of Electrical and Computer Engineering, Neuroengineering Research Initiative and Applied Physics, Rice University, Houston, TX, United States; ^7^Bio-Imaging Lab, University of Antwerp, Antwerp, Belgium; ^8^Max-Planck Institute for Biological Cybernetics, Physiology of Cognitive Processes, Tübingen, Germany

**Keywords:** hV5/MT+, RDK, visual cortex, fMRI, motion coherence

## Abstract

Random dot kinematograms (RDKs) have recently been used to train subjects with cortical scotomas to perform direction of motion discrimination, partially restoring visual motion perception. To study the recovery of visual perception, it is important to understand how visual areas in normal subjects and subjects with cortical scotomas respond to RDK stimuli. Studies in normal subjects have shown that blood oxygen level-dependent (BOLD) responses in human area hV5/MT+ increase monotonically with coherence, in general agreement with electrophysiology studies in primates. However, RDK responses in prior studies were obtained while the subject was performing fixation, not a motion discrimination condition. Furthermore, BOLD responses were gauged against a baseline condition of uniform illumination or static dots, potentially decreasing the specificity of responses for the spatial integration of local motion signals (motion coherence). Here, we revisit this question starting from a baseline RDK condition of no coherence, thereby isolating the component of BOLD response due specifically to the spatial integration of local motion signals. In agreement with prior studies, we found that responses in the area hV5/MT+ of healthy subjects were monotonically increasing when subjects fixated without performing a motion discrimination task. In contrast, when subjects were performing an RDK direction of motion discrimination task, responses in the area hV5/MT+ remained flat, changing minimally, if at all, as a function of motion coherence. A similar pattern of responses was seen in the area hV5/MT+ of subjects with dense cortical scotomas performing direction of motion discrimination for RDKs presented inside the scotoma. Passive RDK presentation within the scotoma elicited no significant hV5/MT+ responses. These observations shed further light on how visual cortex responses behave as a function of motion coherence, helping to prepare the ground for future studies using these methods to study visual system recovery after injury.

## Introduction

Visual motion perception enables us to navigate the environment and avoid collisions with obstacles. In humans, a cardinal area for motion perception is located in the posterior bank of the superior temporal sulcus (STS) in the dorsal middle temporal cortex (area hV5/MT+) (Huk et al., 2002; Becker et al., 2008; Helfrich et al., 2013).

Random dot kinematograms (RDKs) have been used extensively to study the spatial motion integration properties of visual areas of macaques (Newsome and Pare, 1988; Britten, 1998; Rees et al., 2000) and humans (Rees et al., 2000; Vaina et al., 2001; Muckli et al., 2002; Saionz et al., 2020). RDK stimuli force the visual system to extract the global coherent direction of motion from local motion signals that have to be integrated over space and time (Braddick, 1974; Newsome and Pare, 1988; Watamaniuk et al., 1993; Scase et al., 1996) before motion direction can be perceived. The strength of the motion signal of an RDK is modulated either by changing the fraction of dots that move in the same direction (coherence) among a background of randomly moving dots (Newsome and Pare, 1988) or by narrowing the range of directions of motion that each dot can take from frame to frame (Huxlin and Pasternak, 2004; Huxlin et al., 2009). In translational studies, RDKs are being used for rehabilitation of visual motion perception following visual system lesions and in particular to study the recovery of direction of motion perception following primary visual cortex (V1+) lesions (Huxlin et al., 2005, 2009; Cavanaugh et al., 2019; Barbot et al., 2020; Saionz et al., 2020). It is important to study the response of visual areas to RDK stimuli in healthy humans, as well as in subjects with cortical scotomas at baseline, prior to training, in order to better understand how global motion integration is processed in patients versus normal controls. Furthermore, it is important to study RDK processing under two different conditions, that is, when subjects perform a task related to motion discrimination versus a motion-unrelated task at fixation, as task performance is known to modulate cortical area responsiveness (Huxlin et al., 2009).

Electrophysiological studies in macaques (Allman and Kaas, 1971; Dubner and Zeki, 1971; Rodman et al., 1989; Britten and Newsome, 1998; Priebe et al., 2006) demonstrated that the responses of middle temporal (MT) and middle superior temporal (MST) neurons are tuned to the strength of coherent motion (Heuer and Britten, 2007). Specifically, the firing rate of directionally selective neurons in the area V5/MT increases linearly with RDK coherence (Britten et al., 1993) and is correlated with the strength of the monkey’s direction of motion perception (Newsome et al., 1989; Parker and Newsome, 1998). Neurons in other visual areas show variable responses to the coherent motion of RDKs, with early visual areas including V1 tending to show suppression when RDKs are presented over a large field of view. Functional magnetic resonance imaging (fMRI) has been used to measure the responses of human visual areas to motion coherence with less consistent results. Studies on normal subjects show, as expected, higher blood oxygen level-dependent (BOLD) responses for RDK stimuli with high motion coherence in the area hV5/MT+, especially when these are presented over a large field of view (Rees et al., 2000; Braddick et al., 2001; Becker et al., 2008). However, other reports are conflicting, reporting that hV5/MT+ BOLD responses do not depend on (Beer et al., 2002; Smith et al., 2006) or even decrease with motion coherence (McKeefry et al., 1997; Previc et al., 2000). The response of human visual areas other than hV5/MT+ as a function of coherence is less clear. Rees et al. (2000) report that BOLD response is linear as a function of RDK motion coherence in the area hV5/MT+, whereas higher areas (“kinetic occipital,” V3A, frontal cingulate gyrus) tend to show either non-linear U-shaped responses or negative correlation.

Human fMRI studies typically measure the responses of large populations of neurons with diverse properties (Zeki et al., 1991; Tootell et al., 1995) rather than single cells (Kwong et al., 1992) tuned to the direction of motion and may therefore be more sensitive to the overall state of adaptation and to the brain state of the subject while they are performing a task. Task performance, in part through attention, has been known to modulate neural activity in the visual cortex (Azzopardi and Cowey, 2001), specifically in the dorsal stream including hV5/MT+ (Tootell et al., 1995; Heeger et al., 1999; Saenz et al., 2002; Schoenfeld et al., 2007), and it is natural to ask whether the linear response of area hV5/MT+ to motion coherence is also affected. To explore whether RDK BOLD responses depend (1) on the state of adaptation, that is, the baseline visual stimulus condition from which RDK stimuli of various global motion coherence strength are presented (Tolias et al., 2001), and (2) on direction of motion discrimination task performance, we made two protocol modifications: (1) We measured RDK responses as a function of motion coherence when the subject was performing a direction of motion discrimination task versus when they were simply fixating. (2) We measured the response to coherent motion starting from a baseline state elicited by an RDK of zero coherence rather than a uniform gray screen. Starting from this 0% motion coherence baseline allowed us to map responses selective to the spatial integration of coherent motion signals, as opposed to responses induced by the combined changes in local luminance and motion contrast.

Our results under simple fixation starting from a 0% coherence baseline condition corroborated the result that the area hV5/MT+ activity increases as a function of coherence in agreement with Rees et al. (2000). However, when subjects performed the RDK direction-of-motion discrimination task, coherence dependence was essentially abolished. A similar result was obtained in the area hV5/MT+ of subjects with dense cortical scotomas performing direction of motion discrimination for RDKs presented inside the scotoma, whereas passive RDK presentation within the scotoma elicited no significant hV5/MT+ responses. Our results complement the existing literature and help to inform the design of future RDK mapping experiments to study how visual areas reorganize following visual motion perception rehabilitation in subjects with cortical scotomas.

## Materials and Methods

### Human Subjects

The human experiments consisted of three separate RDKs studies.

*Study A:* Six healthy subjects with no history of psychiatric or neurological problems were recruited. Two subjects were excluded from the analysis because of significant head motion in the MR environment (>5 mm), which could not be adequately corrected offline.

*Study B*: For the second study, six more healthy participants who fulfilled our inclusion criteria underwent the experimental procedure.

All the participants for both studies were recruited at the Core for Advanced MR Imaging at Baylor College of Medicine (BCM).

*Study C (patients and controls): Patients:* Seven subjects (27–64 years old, three females and four males) with visual cortical lesions participated in our study. Six of them were recruited at the Max Planck Institute for Biological Cybernetics (MPI) in Tübingen, Germany, and one at the Core for Advanced MR Imaging of the BCM. *Controls:* Six healthy subjects were recruited as control subjects. Four of them were scanned at MPI and two at BCM.

All subjects had normal or corrected-to-normal visual acuity. Experiments were done with the approval of the institutional review board committees of BCM and the Regierungspräsidium of the MPI.

#### Magnetic Resonance Imaging Scans

*For studies A and B:* Functional and structural scans were performed at the Core for Advanced MR Imaging at BCM, using two 3.0-T MRI scanners (Siemens Ltd., Erlangen, Germany): Allegra and TIM Trio, both equipped with a quadrature 12-channel coil.

T1-weighted high-resolution (MPRAGE) scans, for approximately 7 min each, were acquired twice for signal-to-noise ratio (SNR) reduction [repetition time (TR) = 1,900 ms, echo time (TE) = 2.26 ms, matrix size = 256 × 256 × 192, flip angle = 9, spatial resolution Trio/Allegra = 0.5 × 0.5 × 1.0 mm^3^/0.96 × 0.96 × 1.0 mm^3^]. BOLD images were registered onto the anatomical images, which were used to (i) coregister the functional scans to the anatomy of each subject for each session and to (ii) segment each subject’s anatomical data into white and gray matter. Whole-brain T2*-weighted BOLD images were acquired using the single-shot echo planar imaging pulse sequences covering the entire brain (TR = 2,000 ms, TE = 40 ms, matrix size = 64 × 64, voxel size = 3.28 × 3.28 × 3.28 mm^3^, flip angle = 90°, number of slices Trio/Allegra = 28/29).

*For study C:* The scans were performed on a 3.0-T Siemens Prisma (at MPI) and Trio (at BCM) (Siemens Ltd.). For each participant (patient or control subject), two T1-weighted anatomical images were acquired (*Prisma*: voxel size = 1 × 1 × 1 mm^3^, matrix size = 256 × 256 × 192, flip angle = 9°, TR = 2,300 ms, TE = 2.98 ms, TI = 1,100 ms; *Trio*: voxel size = 0.5 × 0.5 × 1.0 mm^3^, matrix size = 256 × 256, 192 partitions, flip angle = 9°, TR = 2,600 ms, TE = 3.5 ms, TI = 1,100 ms). BOLD images were acquired using gradient echo planar sequences with 29 (MPI) and 30 (BCM) contiguous 2.6- and 2.5-mm-thick slices, respectively, covering the whole brain (TR = 2,000 ms, flip angle = 90°, matrix size = 64 × 64, Prisma/Trio TE = 35/30 ms, Prisma/Trio voxel size = 3 × 3 × 2.6/3 × 3 × 2.5 mm^3^). For each subject, five functional scans were acquired, each consisting of 131 image volumes.

#### Stimulus Presentation

*Studies A and B*: Stimuli were projected under photopic conditions onto a rear-projection translucent acrylic screen (DaTex; Da-Lite Corp.) *via* an NEC GT2150 projector (2500 ANSI Lumens, 1,600 × 1,200 resolution, 120 Hz) controlled by a Macintosh computer and seen through an oblique mirror mounted on the MR head coil. The active visual field subtended approximately 15° in radius. When necessary, subjects were corrected for optimal accommodation using magnet-compatible glasses.

*Study C*: At the MPI, for stimulus projection, we used MRI-compatible digital goggles (VisuaStim, Resonance Technology Company, Inc., Northridge, CA, United States), with field of view = 30° (horizontal) and 22.5° (vertical), resolution = 800 × 600, mean luminance 5.95 cd/m^2^. An infrared eye tracker was used to record eye movements (iView XTM; SensoMotoric Instruments GmbH). At the BCM, the stimuli were projected on the same screen as described under studies A and B.

All stimuli were presented under photopic conditions, the dots being dark on the bright background to minimize scattering, and were generated using MATLAB (MathWorks) and the psychophysics toolboxes, Psychtoolbox (Kleiner et al., 2007)^1^ and Vistadisp, an open toolbox (VISTASOFT, Stanford).^2^

#### Stimulus Paradigms

##### Retinotopic Mapping

*Studies A and B:* Retinotopic visual field maps were obtained using phase-encoded retinotopic mapping (Engel et al., 1994) with 45° wedges and 1.5° concentric rings, and the borders of the early visual field areas determined according to Wandell et al. (2007). Stimuli were circular with a maximum radius of 12°. A full wedge cycle was completed in 36 s, with a total of six cycles per scan (216 s). For rings, the pattern of the stimulus was moving in repeating cycles from the center to the periphery through eight expansions of 32 s each (256 s). Both rings and wedges had flickering checkerboard patterns at 2 Hz, spatial frequency approximately 1 c/deg, and 100% contrast, and were acquired with TR 2 s. Two to five scans were performed in each stimulus condition (wedge and ring), depending on the subject.

*Study C:* The stimulus consisted of moving square-checkerboard bars (100% contrast) within a circular aperture with a radius of 11.25° around the fixation point. The bar width was 1.875° and traveled sequentially in eight different directions, moving by a step half of its size (0.9375°) every image volume acquisition (TR = 2 s). The subjects’ task was to fixate on a small dot in the center of the screen (radius: 0.0375°; two pixels) and respond to the color change (red to green) by pressing a button. The color was changing randomly with a frequency of one every 6.25 s.

##### Functional Localizer for hV5/MT+

Area hV5/MT+ was identified using 18-s blocks that alternated between moving (100% coherence) and stationary dot patterns. To reduce adaptation, the direction of the moving dots changed by 45° counterclockwise. Each scan consisted of either six or eight blocks of moving and static dots, depending on the subject. Each scan’s moving and stationary dot patterns were repeated six to eight times during each functional localizer scan. A total of 48 blocks of moving and static dots were acquired for each subject.

Please note that normal retinotopic mapping based on clusters of activity at the expected anatomical locations was also sufficient for identifying hV5/MT+ and provided a second method for ensuring accurate hV5/MT+ identification. No localizer was used for the scans in study C.

##### Motion Coherence Paradigm

*Study A* (subjects did not perform direction of motion discrimination)

Task: During study A, the subjects were instructed to perform a fixation task that did not involve direction of motion discrimination while passively viewing full-field RDKs with different motion coherence. A small dot of 0.15-degree radius was displayed at the center of the visual field to serve as a fixation mark. The color of the fixation point changed between green and red at random times, and the subjects were required to report the color change by button press. All subjects responded correctly for > 96% of fixation color changes, with average response time < 0.6 s.

**FIGURE 1:**
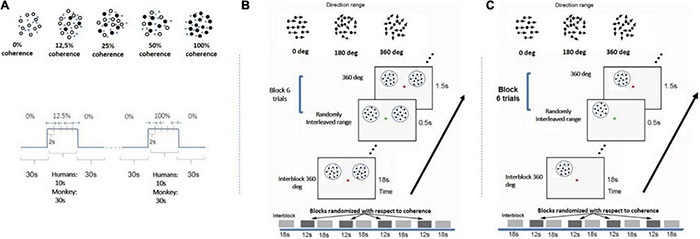
Experimental design. **(A)** Paradigm used in study A. Each motion coherence was presented for 10 s interleaved with 30 s of 0% coherence (no correlation among dots). All coherence levels appear for the same total time in each scan. Each scan either consisted of two blocks of each of four pseudorandomly interleaved coherence levels (12.5, 25, 50, 100%) or four trials of each of three pseudorandomly interleaved coherence levels (25, 50, 100%). Within each block with non-zero coherence, the global direction changed (left vs. right) every 2 s to minimize adaptation. A similar paradigm was used for the monkey experiment except RDK duration was 30 s for all coherences. Black dots represent the group of dots moving in the same direction. **(B)** RDKs for study B were presented at bilaterally symmetric locations (see section “Materials and Methods”). Coherence alternated between 18 s of baseline of 360° direction-range (no global coherence) followed by 1 s of RDK coherence selected from levels (0°, 180, 288°, 360°). All coherence levels were pseudorandomly interleaved and balanced across blocks and scans. Within blocks, coherent RDKs were presented for 0.5 s for six trials (see section “Materials and Methods”) and directions of motion changed every 2 s, with left and right directions appropriately balanced but presented at random sequence (so subjects could not predict the direction). Subjects were fixating and cued by a change in the color of the fixation spot to report the direction of the stimulus in one hemifield. **(C)** A similar paradigm to B except now RDKs were presented unilaterally. Motion-discrimination versus no-motion-discrimination sessions were interleaved in the scanner.

*Stimulus:* Dynamic RDKs were generated within a circular aperture (12-degree radius) using the method described by Newsome and Pare (1988). RDK dots were dark, at a density of 2 dots/degree^2^ and radius of 0.1 degree, and were presented on a gray isoluminant photopic background to minimize light scattering. The random dot pattern was refreshed every 50 ms. A random fraction of the dots was displaced by 0.2° in the same direction (left or right) at a rate of 4°/s, while the remaining dots were replaced by the same number of new dots at random positions. The percent of dots moving in the same direction, that is, the strength of coherent motion signal, varied between 12.5, 25, 50, and 100% in a randomly counterbalanced fashion. Each coherence was presented for 10 s interleaved with 30 s of baseline stimulus at 0% coherence (no correlation among dots). All coherence levels appear for the same total time within each scan. To help subjects maintain a stable level of adaptation to the baseline (0% coherence) stimulus, the 0% coherence stimulus remained on during the whole RDK experiment, except when stimuli with non-zero coherence levels were displayed. Each scan consisted of either two blocks of each of four pseudorandomly interleaved coherence levels (12.5, 25, 50, 100%; subjects 1–3) or four blocks of each of three pseudorandomly interleaved coherence levels (25, 50, 100%; subject 4). Eight (for subject 4) or 10 (for subjects 1–3) scans were performed in a single session (Figure 1A).

*Study B* (bilateral stimulation; unilateral motion discrimination task performance)

Task: During study B, two RDKs were presented at symmetric positions in the left and right visual fields (Figure 1B), respectively, while the subjects fixated at a central spot and performed a motion direction discrimination task on the right RDK as instructed. Study B was carried out to investigate whether performing an RDK-related task changed the shape of the BOLD response profile as a function of motion coherence.

*Stimulus:* RDK stimuli for this experiment were derived according to Huxlin and Pasternak (2004) and Huxlin et al. (2009) The parameters were identical to the ones described previously, except that each dot moved following the same rule, and the global motion strength is modulated by means of the choice of the direction range of each dot (Huxlin et al., 2009; Cavanaugh et al., 2015; Saionz et al., 2020). In this design, each dot randomly “picks” a direction evenly distributed around a central direction (left or right moving). For example, when the range of motion for each dot is 360° (2π), the net global directional motion signal is zero. On the other extreme, when the range of motion is 0°, all the dots move toward the same direction, and net global motion is full strength.

The stimuli were two RDKs 6° in diameter, one (task-relevant) centered at (5, 4) and the other (task irrelevant) at (−5, 4), or *vice versa*. A dot of 0.2 degree in diameter was presented at the center to serve as a fixation spot, as well as instruction cue. Each run consisted of alternating passive fixation period (18 s) and active motion discrimination period (12 s) in the right visual field. During the active motion discrimination period, the fixation spot changed its color from red to green every 2 s, with green indicating the occurrence of global motion (leftward or rightward) that lasted for 0.5 s. The subjects had to report the direction of the perceived motion in the RDK presented in their right visual field by button pressing. For each 12-s period, the motion strength presented in each trial remained the same, whereas the direction of motion varied randomly (leftward or rightward in a balanced fashion). The direction of motion in the left and right RDKs were uncorrelated, but the motion strength (coherence) in both presented RDK patches was the same. Four motion strength levels were tested (360°, 288°, 180°, 0°). During the passive fixation period (RDKs at 0% motion coherence), the fixation spot remained red, and subjects were instructed to passively fixate only, while the stimuli varied identically to the active case. For each scan, each motion strength level was repeated twice, making the total duration of the run 258 s. Each subject underwent 10 scans per session, resulting in 20 repeats per motion strength level.

*Study C* (unilateral stimulation; motion discrimination versus fixation task)

*Task:* Study C consisted of an active and a passive task. For both, the subjects had to fixate a dot at the center of the screen. In the fixation task, the color of the fixation dot changed from red to green at random intervals, and the participants had to respond any time there was a change. In the motion discrimination task experiment, participants were still instructed to fixate the dot, but this time, they had to use their peripheral vision to assess the direction of motion of the RDK stimulus. Whenever there was a change in the color of the central dot, they were asked to indicate the motion direction of the stimulus (left or right).

*Stimulus:* The RDK stimulus used here was similar to study B, except that RDK dots were moving slightly faster at 10°/s (vs. 4°/s in B); the RDK aperture was presented unilaterally, either at the left or right upper quadrant of the visual field (counterbalanced), centered at 4° from the vertical meridian and 3° or 4° above the horizontal meridian with an aperture diameter of 4° or 5°, respectively (for the controls) (Figure 1C).

For each patient, the aperture location and diameter were adjusted in order to fall within their visual field scotoma (S15: center = [5°, 4°], diameter = 5°, S29: center = [5°, 4°], diameter = 6°, S12: center = [5°, 4°], diameter = 7°, V1003: center = [5°, 4°], diameter = 5°, S07: center = [7°, 4°], diameter = 5°, S04: center = [8°, 4°], diameter = 3°, S02: center = [3°, 3°], diameter = 5°, where [*x*, *y*] = *x*° from the vertical meridian and *y*° from the horizontal meridian). Four motion coherence levels (360°, 288°, 180°, 0°) were presented as in study B, where, again, the direction of motion range at 360° corresponded to the baseline condition (no coherence).

For the control subjects, the aperture was presented either at the left or right upper quadrant of the visual field and was centered at 4° from the vertical meridian and, depending on subject, 3° or 4° from the horizontal meridian with an aperture diameter of 4° or 5°, respectively.

*Selecting dot speed*: Regarding the selection of dot speed, we followed the parameters used by Newsome and Pare (1988). In their article, Figure 4 shows that maximum discriminability occurs for dot speeds ranging from 4°/s to 12°/s. Both dot speeds we chose (4°/s for studies A and B and 10°/s for study C) fall squarely within this range. Interestingly, there appears to be a disparity between the dot speed required for optimal behavioral performance and the speed at the peak of the MT neuron tuning functions (the latter is higher). For our study, we chose the dot speeds that favor optimal behavioral performance. The fact that MT neuron tuning functions are fairly broad (e.g., Maunsell and Van Essen, 1983; Britten and Newsome, 1998) and their response to RDK stimuli requires spatial summation, in part, may explain the observed disparity.

##### Patients’ Anatomical Lesions and Visual Field Tests

**FIGURE 2:**
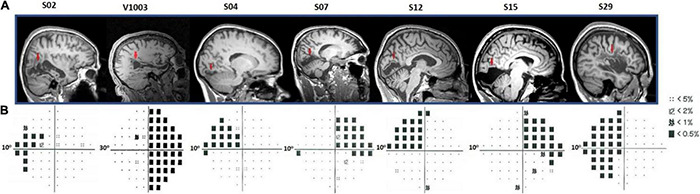
Humphrey’s visual field perimetry test and lesions’ location. **(A)** Sagittal anatomical planes of patients’ brains. The red arrows point to the location of individual’s anatomical lesions. **(B)** Subjects S02, S04, S07, S12, S15, and S29 underwent a 10-degree (10-2) and V1003, a 30-degree (30-2) Humphrey’s perimetry test. The black squares in the pattern deviation probability plots correspond to *p* < −20 dB. Dotted squares correspond to < −10 dB of visual sensitivity. The rest of the locations (small black dots) indicate the non-affected (normal) visual field.

All patients had ischemic or hemorrhagic strokes 7–10 years prior to enrollment. These resulted in dense (visual sensitivity < −20 dB) homonymous visual field deficits within either one or two quadrants of the visual field. MRI anatomical images confirmed the location and extent of the injuries (Figure 2A). In more detail, patient S02 had a left temporal and partial parietal optic radiation injury due to an infarct of the right midposterior temporoparietal lobes. Patients V1003 and S29 had right and left homonymous hemianopia, respectively. Patient S04 had a lesion in the right hemisphere that involved part of the foveal V1v, V2v, and V3v, resulting in a dense left upper visual field quadrant scotoma. The lesion of patient S07 was located in the left inferior calcarine cortex, involving areas left V1v and V1d, left V2v, left V3v, and left V4, resulting in a right homonymous superior quadrantanopic defect. Patient S12 had a lesion in the right inferior calcarine sulcus, involving part of area V1 and visual areas V2v and V3v. Finally, patient S15 had a left temporal optic radiation infarction causing a dense right upper visual field quadrant defect. V1 gray matter remained intact, but a part of it lost its input.

Patients S02, S04, S07, S12, S15, and S29 visual field defects were assessed with a Humphrey-type (10-2) visual field test (Beck et al., 1985; Trope and Britton, 1987) with a (low photopic) background luminance level of 10 cd/m^2^ (Figure 2B). The visual field defects of these patients were also verified using a binocular semiautomated 90° kinetic perimetry obtained with the OCTOPUS 101-perimeter (HAAG-STREIT, Koeniz, Switzerland) (Hardiess et al., 2010). Patient V1003 underwent a Humphrey-type (30-2) visual field test.

### Monkey Experiment

We mapped motion coherence responses in the area V5/MT+ of one rhesus macaque that was trained to fixate. Here, instead of the BOLD signal, we measure modulations in cerebral blood volume (CBV) as a function of RDK motion coherence. To this end, before each scanning session, we injected the monkey with 10 mg/kg MION (monocrystalline iron oxide nanoparticle) (Leite et al., 2002; Leite and Mandeville, 2006). Whole-brain images (TR = 2,000 ms) at 1-mm isotropic resolution using a four-channel phased-array surface coil, with the AC88 gradient insert to increase the spatial resolution, were acquired at 3 T (Siemens Trio at Charlestown Facility, Massachusetts General Hospital, Boston, MA, United States). Fifty slices were acquired using a GE-EPI-T2* functional imaging sequence at TE/TR = 19/2,000 ms, 96 × 84 matrix, 90° flip angle. The monkey was required to fixate, while its eye movements were monitored, using an ISCAN Infrared eye tracker (ISCAN, Burlington, MA, United States).

*Stimulus:* An RDK was constructed as described above (study A) according to the method of Newsome and Pare (1988) with a dot density of 1.7/degree^2^ and dot size (radius) 0.1 degree. This was presented through a central field-of-view ring-like aperture extending from 1 to 12 degrees. The monkey maintained fixation within a 2 × 2 degree fixation window. Dots were dark on a bright background to minimize scattering. Coherent dot displacement was 0.2° every 50 ms in the horizontal direction, while the direction of the dots was reversed every 5 s to minimize adaptation. A 0% coherence background of RDKs was presented for 30 s, alternating with 30 s of RDK stimuli of different motion coherences (12.5, 25, 50, 100%), pseudorandomly interleaved in a block design (Figure 1A). The mean percent BOLD signal modulation greater than the 0% coherence condition was computed for the retinotopically corresponding portions of the V1, V2, V3, V3A, V5/MT, FST, and MST areas.

## Analysis

*Studies A and B:* FMRI data were preprocessed in AFNI (Cox, 1996) and then analyzed with custom MATLAB codes. Segmentation of gray from white matter was performed, and cortical surface reconstruction was carried out with FreeSurfer^3^ using the high-resolution T1-weighted images obtained in the Trio/Prisma scanners. The surface reconstruction algorithm removed extracerebral voxels *via* a skull stripping routine, which results in an intensity-normalized image. Raw functional data were first corrected for slice acquisition time difference, and then motion correction was performed to align all EPI images to the EPI image acquired closest to the T1-weighted images. The fMRI signal time course was detrended to remove slow linear drifts of the fMRI signal. For retinotopic mapping and hV5/MT+ localization, data from scans under the same stimulus condition were averaged together after preprocessing and before data analysis in MATLAB. The averaged time series were then mapped to the smoothed white matter surface using 3dVol2Surf in AFNI, which generates the value for each surface node by transforming the original to standard-mesh surfaces (Saad and Reynolds, 2012). The reference hemodynamic response function used to convolve the stimulus profile is a gamma function *t^b^* exp(*−t/c*), with *b* = 8.6 and *c* = 0.547 s.

Statistical analysis was performed on the fMRI data in the RDK experiments using a generalized linear model (GLM) approach. For each coherence level, a regressor was generated by convolving the stimulus profile of that coherence with the hemodynamic response function above. A third-order polynomial was used to model slow baseline shift. The data from different scans were concatenated for the GLM analysis. The model parameters were estimated using the 3dREMLfit program in AFNI, which uses an ARMA(1,1) model to estimate the correlation structure in noise. After GLM analysis, regions of interest (ROIs) were defined as clusters with corrected *p* < 0.05 across all coherence levels, and that overlap with different visual areas as determined *via* retinotopy was identified. We calculated BOLD time-series averages in identified ROIs as follows: (i) The mean ROI time series was computed by averaging the BOLD signal across the voxels belonging to each ROI and over trials presented at the same coherence level; (ii) the baseline BOLD signal was computed from the last five time points of the baseline stimulus, plus the first time point after the transition (before the BOLD signal has had time to rise).

*Retinotopic mapping and MT localizer:* For retinotopic mapping, the measure of coherence^4^ of the average time course at the stimulus frequency is used as a measure of the BOLD response strength (VISTASOFT, Stanford, see text footnote 2). To locate activated voxels in hV5MT+ localizer scans, we calculated the correlation coefficient between the time courses of the BOLD signal from each voxel and the stimulus time course and assessed significance using the *t*-test. Voxels with significantly different *t*-values from zero define the area hV5/MT+. Putative area MST can be identified as the voxels activated by both contralateral and ipsilateral RDK stimuli, but largely overlaps with area hV5/MT, so we designate both areas together as hV5/MT+.

*Study C:* The functional images were corrected for motion in between and within scans (Nestares and Heeger, 2000) and aligned to the high-resolution anatomical volume using a mutual information method (Maes et al., 1996). We performed the preprocessing steps, in MATLAB using the VISTASOFT toolbox (see text footnote 2).^4^ We fitted a GLM to the time course of each voxel to estimate the contribution of each direction range stimulus tested to the time course. The four conditions tested (direction range: 0°, 180°, 288°, 360°) were then contrasted against the baseline (interblock, direction range: 360°) to estimate the dependence of each voxel on coherence. Only those voxels for which the linear model explained more than 3% of the variance in the data were retained. This threshold was set after measuring the mean explained variance during the passive task in a non-visually responsive area by selecting an ROI (i.e., a sphere of 1 cm diameter) from the lower medial prefrontal cortex and setting the value of the threshold at 3 standard deviations above the mean. In an ROI, the percentage signal change was calculated by averaging the β weights of each predictor for each voxel.

*Retinotopy:* We identified area hV5/MT+ using the population receptive field (pRF) mapping method (Dumoulin and Wandell, 2008). In short, the implementation of the pRF model is a circularly symmetric Gaussian receptive field in visual space. The center and radius of the pRF are estimated by fitting the BOLD signal responses to estimated responses elicited by convolving the model with the moving bar stimuli. We retained only those voxels in these visual areas, for which the topography explained more than 12% of the variance. This threshold was set after measuring the mean explained variance (6 ± 2%) in a non-visually responsive area by selecting an ROI (i.e., a sphere of 1-cm diameter) from the lower medial prefrontal cortex and setting the value of the threshold at 3 standard deviations above the mean.

*Reconstruction of the lesioned hemisphere method*. Analyzing the functional data of patients with cortical visual lesions can be tricky because of the lack of cortical tissue in the location of the injury. To overcome this, we used a method we developed earlier, and it is described in more detail by Papanikolaou et al. (2019) in order to create a “hybrid” hemisphere. In brief, the method uses information from the healthy hemisphere in order to reconstruct the damaged part.

## Results

### Normal Subjects

#### Random Dot Kinematogram Stimulus Presentation Without Motion Discrimination (Study A)

**FIGURE 3:**
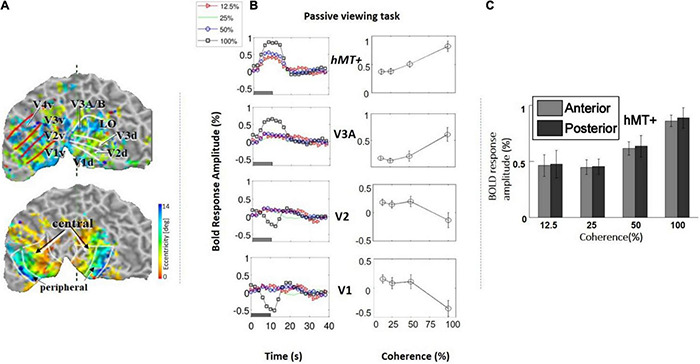
**(A)** *Top:* Example of a retinotopic map presented on the flattened visual cortex of a subject. The lines define the borders between visual areas. *Bottom:* Example of an eccentricity map. The foveal (central) and peripheral ROIs in V1, V2, and V3 are indicated by the black arrows. **(B)** *Left:* Average BOLD signal intensity responses as a function of time in four subjects across four motion coherence levels (12.5, 25, 50, 100%) in visual areas V1, V2, V3A, hV5/MT+, while fixating (no motion discrimination task). The baseline BOLD activity elicited by 0% coherence was subtracted (see section “Materials and Methods”). *Right*: BOLD response amplitude as a function of motion coherence (see section “Materials and Methods”) in the passive viewing condition, across four subjects. Error bars represent the standard error of the mean. **(C)** Selecting the anterior and posterior voxels of hV5/MT+ did not show any difference in coherence dependence. Therefore, we grouped them together for analysis as the hV5/MT+ complex.

**FIGURE 4:**
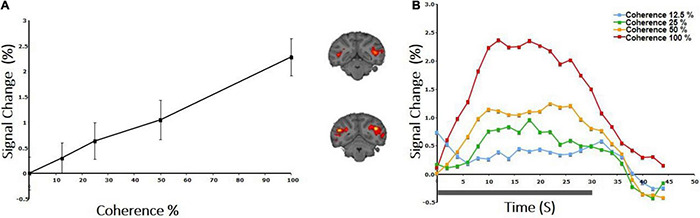
CBV response to coherence in the area V5/MT+ of a rhesus macaque. **(A)** Both area V5/MT and MST behaved in a similar manner and are grouped together. Note the monotonic increase of the CBV signal as a function of coherence. **(B)** BOLD signal intensity modulation induced by presenting an RDK with motion coherence levels: 12.5, 25, 50, 100%. Transition from the baseline (0% coherence) occurred at time 0, and the duration of the coherent RDK presentation was 30 s.

We measured the average BOLD signal modulation across four levels of motion coherence (12.5, 25, 50, and 100%) in areas V1, V2, V3, and V4, and hV5MT+ as a function of time following a transition from 0% coherence. RDKs were generated using the method of Newsome and Pare (1988). Subjects were asked to fixate and report the change of color of a dot at fixation (see “Materials and Methods”), performing > 96% correct in this task. As expected, BOLD signal in the area hV5/MT+ (Figures 3B,C) showed strong statistically significant coherence dependence [*F*(3,11) = 6.21 *p* = 0.01], one-way analysis of variance (ANOVA) over coherence across subjects, each measurement reflecting the mean BOLD response amplitude averaged across trials at the same level of coherence for each subject). Area hV5/MT+ BOLD response increased with coherence, reaching approximately 0.5% greater than baseline at 100% motion coherence. Area V3A was also significantly modulated by coherence [Figure 3B; *F*(3,11) = 7.21, *p* < 0.006, one-way ANOVA], showing a strong increase in BOLD signal intensity at 100% motion coherence and weaker responses at lower motion coherence levels. In contrast, areas V3 and V4 were not significantly modulated by motion coherence levels [*F*(3,11) = 0.54, *p* = 0.66], and [*F*(3,11) = 1.59, *p* = 0.2, respectively, one-way ANOVA]. As reported before (Braddick et al., 2001), the BOLD response in area V1 decreased at higher motion coherences compared with the 0% coherence baseline. Specifically, following a transition from 0 to 100% coherence, the BOLD signal in area V1 decreased, reaching minimum approximately 10 s after the transition [Figure 3B_bottom; *F*(3,11) = 6.90, *p* < 0.01, one-way ANOVA]. Area V2 showed a similar trend that did not reach significance [Figure 3B; *F*(3,11) = 2.79, *p* = 0.09, one-way ANOVA]. These results were qualitatively similar to results obtained in monkey visual cortex during passive fixation, using CBV imaging with MION (Mandeville and Marota, 1999; Smirnakis et al., 2007). Monkey V5/MT showed a clear monotonic increase of CBV signal as a function of coherence (Figure 4). Area MST behaved similarly. CBV versus motion-coherence profiles computed for areas V1, V2, V3, V3A, and FST were, in general, less sensitive to coherence, displaying similar features as the corresponding areas in the human.

**FIGURE 5:**
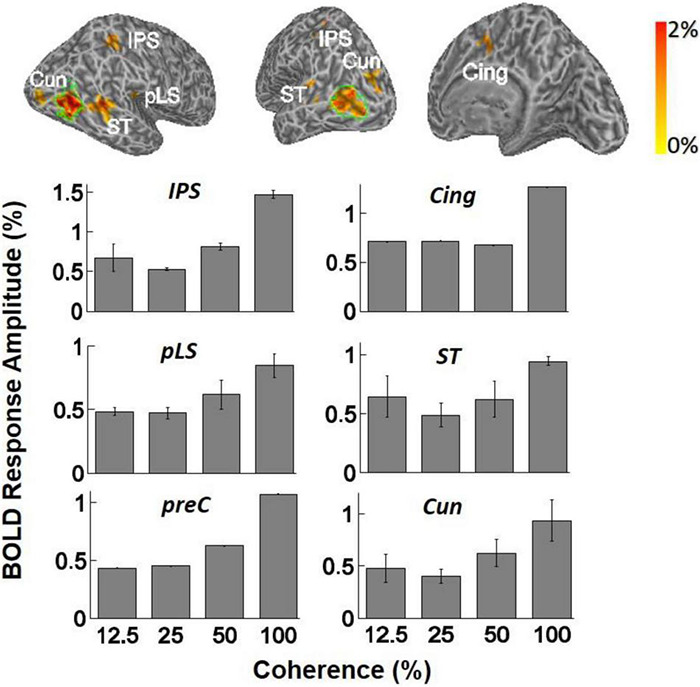
*Top:* Examples of inflated white matter surfaces of individual subjects. Overlaid color maps represent the mean response amplitude across coherence levels of 25, 50, and 100% and show only the region with corrected *p*-values less than 0.05 in the GLM analysis. The green contours delineate the boundaries of area hV5/MT+ as determined from the hV5/MT+ localizer scans. Labels denote the anatomical locations of activated areas: ST, superior temporal sulcus; pLS, posterior lateral sulcus; Cun, cuneus; IPS, intraparietal sulcus; Cing, cingulate cortex; PreC, precentral sulcus. *Bottom:* Averaged fMRI response amplitudes in the activated brain areas (ST, IPS, Cing, PreC, pLS, and Cun) across subjects. The mean BOLD magnitude increases significantly as a function of motion coherence. Only subjects that showed significant activation in these areas when we contrasted all-coherent RDKs against the baseline are averaged (see section “Results for a Discussion”). Note that the analysis used the corrected-for-multiple-comparisons *p*-values for identifying which voxels were activated and then simply reported the responses inside the areas where the activation occurred. We did not intend to compare the level of activity across these areas. In any event, this should be considered as an exploratory study, identifying coherence modulated areas for further study in the future.

BOLD signal specific to motion coherence was present also in higher areas, as determined by contrasting all epochs of motion coherence against the 0% coherence baseline. Significant activation was observed in the cuneus (Cun) in four of four subjects [*F*(3,11) = 3.64, *p* < 0.05, one-way ANOVA], the STS in three of four subjects [*F*(3,11) = 3.61, *p* < 0.05, one-way ANOVA], the posterior lateral sulcus (pLS) in 3/4 subjects [*F*(3,11) = 3.90, *p* < 0.05, one-way ANOVA], and the intraparietal sulcus (IPS) in two of four subjects [*F*(3,11) = 11.56, *p* = 0.001, one-way ANOVA]. In STS, pLS, and IPS, the mean BOLD magnitude increased as a function of motion coherence, similar to hV5/MT+. Each ANOVA measurement reflects mean response amplitude across trials at the same coherence level (Figure 5). Visual motion-related activation in these areas has been observed in earlier studies (Sunaert et al., 1999), except perhaps for pLS. We also observed significant modulation with coherence in the cingulate (Cing) and precuneus (preC), but this was for only a single subject (one of four).

Results presented so far were obtained with passive stimulus presentation and did not involve performing a direction of motion discrimination task. In what follows, we compare visual responses while subjects are performing an RDK-dependent direction of motion discrimination task versus a luminance modulation task at fixation.

#### Performing Direction of Motion Discrimination Flattens Motion-Coherence Dependence in hV5/MT+

**FIGURE 6:**
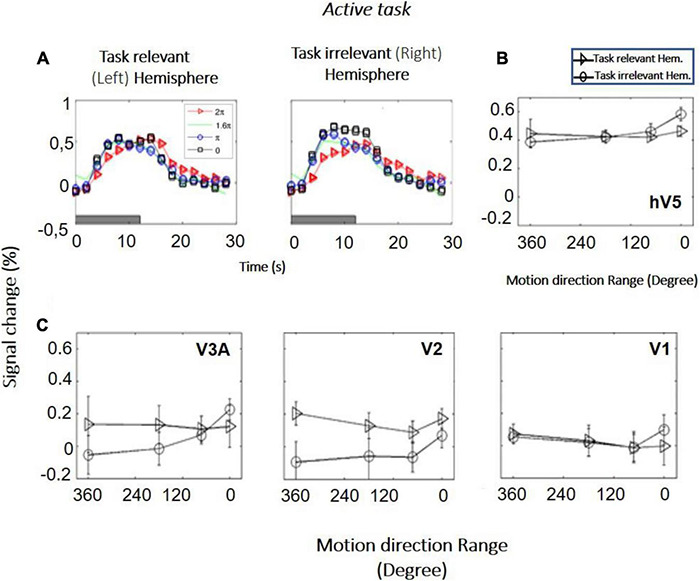
BOLD activity as a function of coherence in the task-relevant versus the task-irrelevant hemisphere (study B). **(A)** BOLD signal change in hV5/MT+ of the task-relevant (left) versus the task-irrelevant (right) hemisphere of one subject. **(B)** Average BOLD signal change in the area hV5/MT+ of the task-relevant (left, triangle) versus the task-irrelevant (right, circle) hemisphere across 6 subjects. **(C)** Same as B for areas V3A, V2, and V1. Error bars indicate the standard error of the mean across subjects.

*Study B (bilateral stimulation):* Six additional subjects were tested with RDKs that were simultaneously presented in symmetric locations, one in each hemifield. Subjects were asked to fixate versus to perform a direction of motion discrimination task in one of the hemifields. RDKs for this task were generated using the range-of-directions method introduced by Huxlin and Pasternak (2004), as we wanted to test coherence responses with the same stimuli used in visual rehabilitation (Huxlin et al., 2009). The reason that in study B we changed from the RDKs of Newsome and Pare (1988) to the range-of-motion RDKs used by Huxlin and Pasternak (2004) was to ensure our observations remain robust under different RDK conditions and specifically for the type of RDK stimulus more commonly used for visual rehabilitation (Huxlin et al., 2009). Four different coherence levels (range of motion: 0°, 180°, 288°, 360°) were tested, presented from a baseline range-of-motion condition of 360° (no global motion coherence). As expected, both the left and the right hV5/MT+ complexes exhibit increased modulation during epochs of stimulus presentation (Figures 6A,B). This was true even when the stimulus presented was the 360° RDK, which is identical to the baseline. This indicates that in this case the BOLD response does not reflect the characteristics of the stimulus itself, but rather stimulus anticipation and/or task-related demands (subjects were cued to respond to a new stimulus presentation by a change of color in the fixation spot; see section “Materials and Methods”). We applied one-way ANOVA, as we wanted to test if the BOLD signal response as a function of coherence differed between the “task-relevant” versus the “task-irrelevant” hemisphere. Interestingly, we found such a difference. In particular, hV5/MT+ BOLD response was flat as a function of coherence [*F*(3,20) = 0.45, *p* = 0.71] in the task-relevant hemisphere (the hemisphere contralateral to the RDK whose direction of motion the subject was tasked with reporting). In contrast, hV5/MT+ BOLD response increased as a function of coherence in the task-irrelevant hemisphere [*F*(3,20) = 4.94, *p* = 0.009], as observed in studies A and C (see below).

*Study C (unilateral stimulation):* We used random dot stimuli to characterize responses in hV5/MT+ complex to motion coherence in six additional healthy subjects and seven patients under two conditions. In the first condition (passive task), subjects performed a fixation task at the center of the screen, while RDKs of different coherences were presented unilaterally away from fixation (see section “Materials and Methods”). In the second condition (active task), subjects were instructed to report the direction of motion of RDKs presented at identical locations, while maintaining fixation. RDKs for this task were generated using the range-of-directions method introduced by Huxlin and Pasternak (2004), as in study B. Note that RDKs were smaller and unilaterally presented compared with RDKs in study A (which were 12° in radius and centrally presented).

**FIGURE 7:**
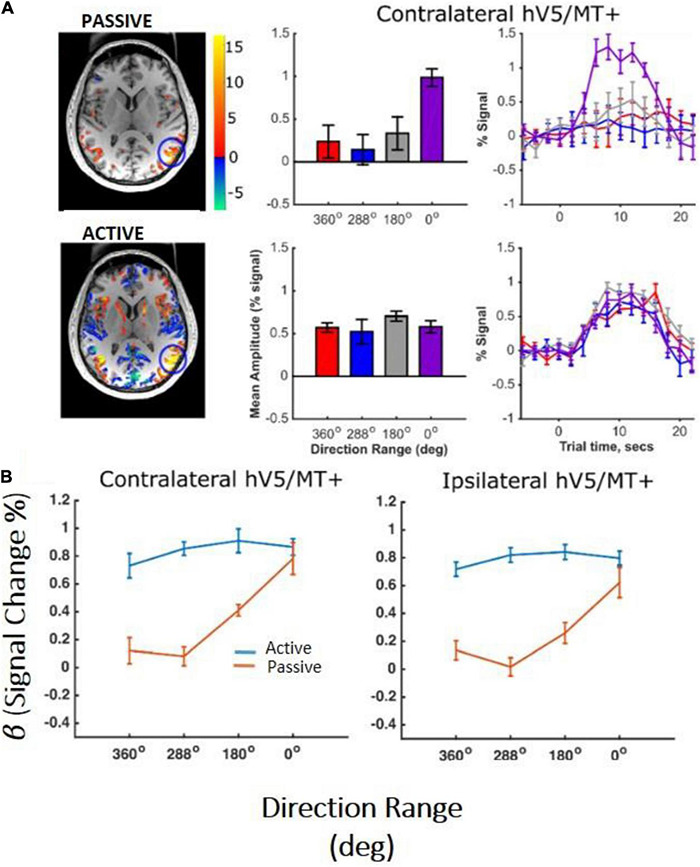
hV5/MT+ responses to motion coherence in control subjects (study C). **(A)** Activation maps and average BOLD signal change in the contralateral hV5/MT+ of one subject, while the subject was performing an active direction of motion discrimination task (bottom) versus an unrelated task (passive) at fixation (top). For the passive condition, our results are consistent with our previous findings and the literature that BOLD signal modulation increases with coherence when no discrimination task is used. However, when the subject is performing direction of motion discrimination, the BOLD signal modulation is approximately the same at all coherences. **(B)** Average % BOLD signal change for all participants (*n* = 6) performing the motion discrimination task (blue line) versus fixation (orange line). Error bars indicate the standard error of the mean across subjects. As in **(A)**, when the subjects are performing the direction of motion discrimination task, the BOLD signal modulation is approximately the same at all coherences. In contrast, when subjects are fixating without performing the motion discrimination task, BOLD signal modulation increases monotonically with coherence level. A similar activation pattern is also observed in ipsilateral hV5/MT+, presumably reflecting voxels that belong primarily to MST whose receptive fields are bilateral.

In the control subjects, the hV5/MT+ complex showed significant activation to motion coherence both in the contralateral hemisphere and in the ipsilateral hemisphere relative to the stimulus presentation. As expected, more voxels were significantly activated in the contralateral hV5/MT+ complex (Figure 7A), whereas voxels activated in the ipsilateral hV5/MT+ complex in part correspond to area MST, whose receptive fields are bilateral. Activated voxels showed strong BOLD signal modulation with coherence both ipsilaterally (presumably corresponding largely to area MST) and contralaterally with respect to the stimulus presentation (Figure 7B). Then, we tested the dependence on coherence when the participants were fixating and when they were performing a motion discrimination task. To check this, we conducted a one-way ANOVA, for both ipsilateral and contralateral hV5/MT+ separately. Similarly to study A, hV5/MT+ increased with motion coherence when the subject was performing a motion-unrelated task at fixation [*F*(3,16) = 6.53, *p* = 0.04, for the ipsilateral hV5/MT, and *F*(3,16) = 8.35, *p* = 0.001, for the contralateral hV5/MT; Figure 7B; orange lines]. However, when subjects were asked to perform direction of motion discrimination, the dependence of the BOLD signal on coherence was markedly suppressed and essentially completely abolished [Figure 7B; blue lines, ipsilateral hV5/MT: one-way ANOVA *F*(3,16) = 0.40, *p* = 0.74, and contralateral hV5/MT: *F*(3,16) = 0.75, *p* = 0.53].

##### Patients With Dense Cortical Visual Field Scotomas

We measured the hV5/MT+ responses to motion coherence for seven subjects with early visual cortical lesions resulting in a dense homonymous visual field scotoma (see section “Materials and Methods”). Three patients (patients S04, S07, and V1003) were tested while performing the passive fixation task. Six patients, two of whom had performed the passive task (S04, V1003), and four other patients (S12, S15, S29, S02), were tested while performing the active task. Patient responses were tested both in the sighted and in the blind visual field.

**FIGURE 8:**
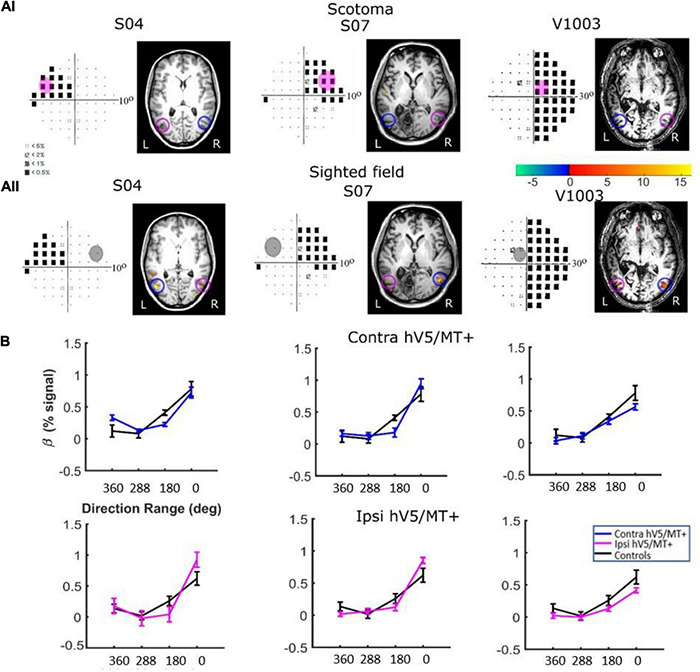
hV5/MT+ responses to motion coherence in three patients during the passive task. **(Ai)** Visual field deficits for each patient based on perimetry and activation maps when the stimulus was presented within the blind visual field (magenta disk). We found no significant activity in either the contralesional (blue circle) or ipsilesional (magenta circle) hV5/MT+ when the stimulus was presented within the patients’ scotoma and the patients were performing a passive fixation task. **(Aii)** Activation maps when the stimulus was presented within the sighted visual field (gray disk). We found significant activity for both the contralesional (blue circle) and ipsilesional (magenta circle) hV5/MT+ when the stimulus was presented in the sighted field of the patients. **(B)** Mean GLM β weights of hV5/MT+ across patients as a function of coherence level when the stimulus was presented within their sighted visual field compared with control subjects (black). Responses in both the contralesional (blue) and ipsilesional (magenta) hV5/MT+ were similar to control subjects.

**FIGURE 9:**
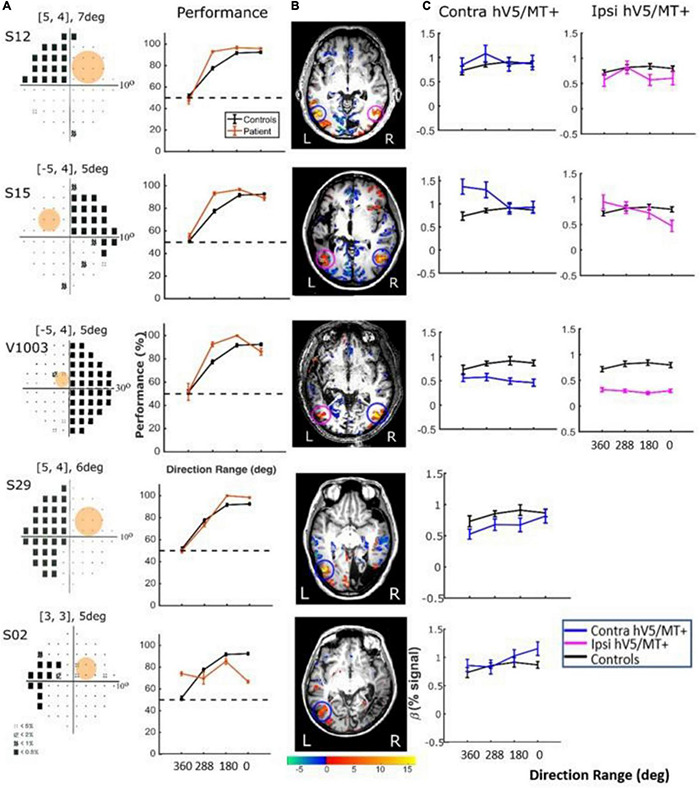
Behavioral performance and hV5/MT+ responses to motion coherence within the sighted field of patients performing a motion direction discrimination task. **(A)** *Left column:* Visual field deficits for each patient based on perimetry and location of the stimulus aperture (orange disk). *Right column:* Behavioral performance for the motion direction discrimination task when the stimulus is presented within the sighted field of patients (orange) versus control subjects (black). **(B)** Activation maps when the stimulus was presented within the sighted visual field. We found significant activity for both the contralesional (blue circle) and ipsilesional (magenta circle) hV5/MT+. **(C)** Mean GLM β weights of contralesional (blue) and ipsilesional (magenta) hV5/MT+ across patients as a function of coherence level compared with control subjects (black).

*Responses in the Sighted Visual Field. P*atients did show significant activation in both the contralesional and ipsilesional hV5/MT+ when the stimulus was presented in their sighted visual field (Figures 8B, 9B). The dependence of BOLD responses in both contralesional and ipsilesional hV5/MT+ to motion coherence was similar to the healthy subjects presented above, that is, linearly increasing with coherence when subjects were performing a task at fixation [a two-way ANOVA, with factor A the two groups and factor B the different coherences, revealed that there was a non-statistically significant difference between the two groups with *F*_*A*_(1,83) = 0.001, *p* = 0.97, while the responses to the coherences were significantly different (tuned): *F*_*B*_(3,83) = 36.18, *p* = 4.663 × 10^–15^; Figure 8B]. On the contrary, a similar two-way ANOVA performed when the subjects were engaged in a direction of motion discrimination task showed non-significant differences between the groups [*F*_*A*_(1,69) = 3.67, *p* = 0.059], and the responses to the different coherences were flat [i.e., not significantly different; *F*_*B*_(3,69) = 1.07, *p* = 0.36 Figure 9C].

*Responses in the Blind Visual Fieldreveal visually driven activation in both ipsilesional.* : Although retinotopic mapping did reveal visually driven activation in both ipsilesional and contralesional hV5/MT+, contrasting coherently moving stimuli to stimuli of 360° direction of motion range, that is, no motion coherence, revealed no significant activation during the passive fixation condition when the RDK stimulus was presented inside the patient’s scotoma (Figure 8Ai). Note that this does not necessarily mean that hV5/MT+ of these patients does not get activated by motion stimuli presented within the scotoma, but rather that the modulation is flat as a function of coherence (i.e., it does not differ significantly from the 360° range coherence baseline). Therefore, we can conclude that hV5/MT+ RDK responses arising from stimuli presented inside the dense cortical scotoma are either too weak to elicit significant modulation and/or do not vary significantly as a function of motion coherence.

**FIGURE 10:**
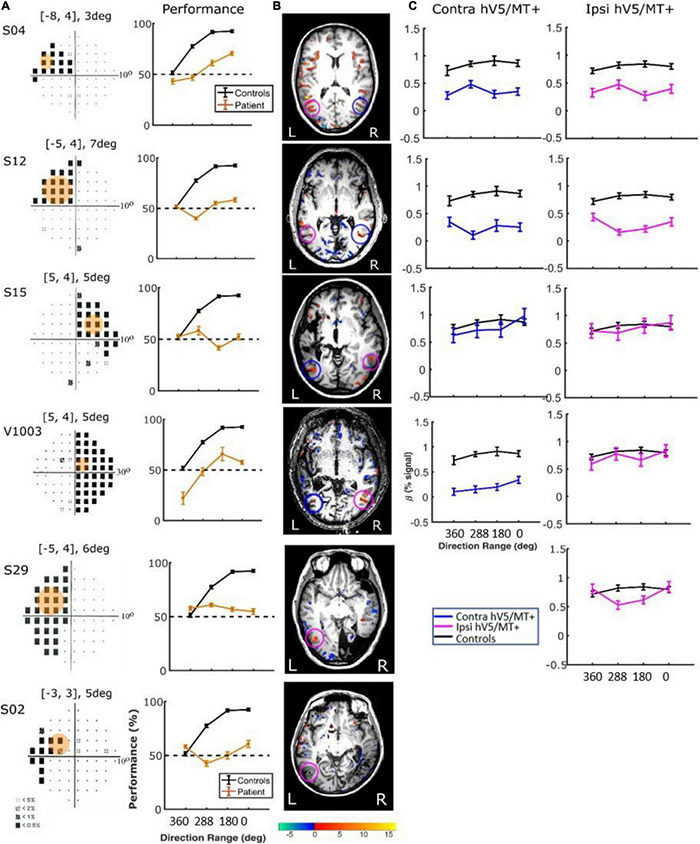
Behavioral performance and hV5/MT+ responses to motion coherence within the blind field of patients performing a motion direction discrimination task. **(A)** *Left column:* Visual field deficits for each patient based on perimetry and location of the stimulus aperture (orange disk). The center coordinates and diameter of the aperture are shown on top of each graph in the form [*x*, *y*], *z*deg, where *x* = deg from vertical meridian, *y* = deg from horizontal meridian, and *z* = deg diameter. *Right column:* Behavioral performance for the motion direction discrimination task when the stimulus was presented within the blind field of patients (orange) versus control subjects (black). **(B)** Activation maps when the stimulus was presented within the blind visual field. We found significant activity for both the contralesional (blue circle) and ipsilesional (magenta circle) hV5/MT+. **(C)** Mean GLM β weights of contralateral (blue) and ipsilateral (magenta) hV5/MT+ across patients as a function of coherence level compared with control subjects (black). The variability observed could relate to differences in the type of lesion that each subject has or differences in the ability of the subjects to cope with the requirements of the active task. The fact that some subjects show responses at the level of sighted controls is consistent with the hypothesis that responses are driven by task-related load or attention.

In contrast, performing the direction-of-motion discrimination within the patients’ scotoma elicited significant responses in hV5/MT+, as judged by contrasting all RDK coherent-motion conditions tested against the baseline (360° direction of motion, i.e., no motion coherence) (Figure 10). The BOLD signal as a function of coherence was suppressed and approximately unchanged at all coherences similar to the responses observed in healthy subjects and patients when the stimulus was presented in their sighted field under the direction of motion discrimination condition. A two-way ANOVA, with factor A the two groups and factor B the different coherences, confirmed that there is a significant difference between the groups [*F*_*A*_(1,76) = 22.01, *p* = 0.00001], while the coherences remain flat [*F*_*B*_(3,76) = 0.86, *p* = 0.46]. This is markedly different than the profile of BOLD signal modulation as a function of coherence during the passive fixation condition when the stimulus was presented in the sighted field. This occurred while the patients’ performance remained at chance at all coherence levels when the stimulus was presented in their blind field, confirming the visual field defect and also suggesting that the subjects did not significantly break fixation (Figure 10A, see section “Behavioral Performance”).

There were some differences across subjects, but these do not change the basic observations reported previously. Specifically, for patients S02 and S29, hV5/MT+ was itself lesioned, and only ipsilateral data could be obtained (all other patients showed bilateral hV5/MT+ activation upon unilateral stimulus presentation). The hV5/MT+ area activated contralateral to the stimulus presentation was significantly smaller than control subjects.

*Behavioral Performance.* We used one-way ANOVA to test the difference of performance in patients versus controls. We found that the performance of the patients was commensurate to control subjects [*F*(3,16) = 1.19, *p* = 0.34; Figure 9A, when the stimulus was presented in the sighted visual field, but there was a significant difference (fall in performance as a function of coherence) between patients and controls [*F*(3,20) = 15.36, *p* = 0.0002], when the stimuli were in the blind field. Two-tailed *t*-tests were also performed to compare the performance of subjects versus controls at each coherence. When there is no coherent motion (direction of motion: 360°) patient performance was commensurate to control performance (two-tailed *t*-test, *p* = 0.60), whereas for all other values (288, 180, 0°), control subjects performed superiorly to patients (*p* = 0.0002, *p* = 0.0001, *p* = 0.0001, respectively, Figure 10A).

## Discussion

This study revisited the question of how human visual cortex responds to motion coherence stimuli. We went further than previous studies in several respects: (1) we demonstrated how BOLD fMRI responses are modulated by task relevance; (2) we used the non-coherent motion condition as baseline instead of a static stimulus in order to maximize specificity to global motion integration; (3) we validated part of our findings in a non-human primate; and (4) we showed that our observations remain consistent in patients with visual scotomas arising due to cortical injury.

Overall, in healthy subjects, passive RDK presentation starting from a baseline condition of 0% coherence resulted in robust activation of the hV5/MT+ complex and area V3A, as well as the higher areas (Cun, pLS, ST, Cing, IPS, preC). The result in hV5/MT+ complex is in general agreement with Rees et al. (2000) and Becker et al. (2008), whose baseline condition involved static stimuli. On the face of it, the amplitude of the BOLD response to 100% coherence in the area hV5/MT+ we observe (approximately 0.5%) appears to be stronger than the one reported by Rees et al. (2000) (0.1%), but this difference can be largely explained on the basis of a difference in the experimental design. Specifically, Rees et al. (2000) used an event-related paradigm with much shorter stimulus presentation periods. Once this difference is taken into account, one arrives at very similar underlying firing rate estimates using the model introduced by Rees et al. (2000). This result is reassuring, as the baseline condition we used, unlike the one selected by Rees et al. (2000) and Braddick et al. (2001) does maximize the chance that the observed BOLD responses are selective for the integration of local motion signals to global motion coherence. It is also consistent with Braddick et al. (2001), who had also used a dynamic moving baseline with 0% coherence obtaining similar results.

On the other hand, our results are in contrast with results by McKeefry et al. (1997) and Smith et al. (2006) who found that the area hV5/MT+ responses were not modulated by motion coherence. We believe that differences in the experimental conditions are the most likely reason for the disagreement in conclusions. Specifically, McKeefry et al. (1997) and Smith et al. (2006) used paradigms with very low RDK dot densities leading to a decreased chance for motion integration within the receptive fields of neurons in hV5/MT+. The BOLD response in each voxel depends not only on the strength of the motion signal but also on the number of different motion directions stimulated within its pRF. When motion coherence increases, the number of different motion directions stimulated decreases. As first pointed out by Braddick et al. (2001), the balance between these two factors changes with dot density. For example, accounting for the eccentricity of our stimuli, we calculated that the average number of moving dots within the typical area of an hV5/MT receptive field would be approximately 50, compared with approximately 4–6 for the stimuli used by McKeefry et al. (1997) and Smith et al. (2006). Therefore, the number of dots in these two studies is much lower than our study and also than that in the studies by Rees et al. (2000); Braddick et al. (2001), and Becker et al. (2008). Hence, when the level of coherence increases, the increased global motion strength may not be able to overcome the decreased number of motion directions that stimulate the typical area hV5/MT voxel. The degree of motion opponency in different areas likely also plays a role.

Another noteworthy finding of our study is that unilateral RDK stimulus presentation modulated both the ipsilateral and contralateral hV5/MT+ (Figure 7B). Although the number of ipsilateral voxels activated in the hV5/MT+ complex was fewer than the contralateral ones, presumably corresponding to area MST whose large receptive fields cross the midline, we found that their coherence dependence was similar to those in the contralateral side. This suggests that coherence dependence is similar for both areas V5/MT and MST. Moreover, in a separate analysis (Figure 3C), separating area hV5/MT+ voxels to anterior and posterior groups did not reveal a difference in coherence dependence (Figure 3C), supporting that there is no strong difference in coherence dependence between area hV5/MT and the more posteriorly located, putative MST. This agrees qualitatively with Becker et al. (2008), who found that the area MST responses increase with motion coherence.

A novel and surprising finding in our study was the suppression of coherence dependence in areas hV5/MT+ and V3A observed when healthy subjects performed a direction of motion discrimination task instead of passive fixation. We conjecture that increased task-related demands and/or allocated attention during the active visual motion discrimination task likely increase disproportionately the BOLD responses in these motion-selective areas for low coherences, thus flattening the coherence dependence (Figures 6B, 7B). BOLD signal responses in our study were increased commensurately even for the non-coherent stimulus condition, which was identical to the baseline, suggesting that they are not specific to global motion integration across space. It is thus probable that coherence dependence is masked by a non-selective effort-related increase in the BOLD signal response within fMRI voxels. Attention to the stimulus or task-related demands during the motion discrimination task would tend to increase the BOLD response in hV5/MT+ (Huk et al., 2001), and this may indeed be the underlying reason for our findings. It remains to be investigated whether ensuring that the load of attention or task-related demands remains the same across different levels of coherence can reinstate the monotonic dependence of hV5/MT+ activation as a function of coherence.

### Motion Coherence Responses in Areas Outside the hV5/MT+ Complex

We also found modulation of the BOLD signal as a function of coherence in higher motion-processing areas, such as the Cun, STS, pLS, and the intraparietal cortex. Others have also documented parietal activation in response to discrimination of motion (Vanduffel et al., 2002; Orban et al., 2004; Peuskens et al., 2004), as well as in the area hV5/MT+ and lateral occipital cortex (Braddick et al., 2000; Grill-Spector et al., 2001; Kourtzi and Kanwisher, 2001). BOLD signal in these areas increased with motion coherence qualitatively similarly to area hV5/MT+ (Figure 3).

In contrast, responses in early visual areas (V1/V2) decrease as coherent motion strength increases (Figure 3B). This finding agrees with earlier reports by Braddick et al. (2001), but contrasts with McKeefry et al. (1997), who found the opposite. Again, this is likely the result of the difference in the density of the dots between our stimuli. At 6° eccentricity, the mean receptive field size of area V1 neurons is 0.53 (Dow et al., 1981), so the average number of dots within a typical V1 receptive field would be approximately 0.5. Therefore, increased coherence level does not enhance the motion signal strength detected by individual V1 neurons. The BOLD response in V1 would then be solely determined by the number of different motion directions that fall over time in the receptive field. As the coherence level increases from 0 to 50%, the density of dots corresponding to random noise changes from 2/degree^2^ to 1/degree^2^, and the number of motion directions stimulating each voxel over time varies relatively little. However, at 100% coherence, the number of motion directions corresponding to random noise is reduced fairly abruptly to zero. This may explain the relative flat dependence of V1 BOLD responses at coherence levels of 12.5, 25, and 50%, versus the prominent negative response at coherence 100% (Figure 3A; passive task). Interestingly, when subjects perform a direction of motion discrimination task, the response to coherence is flat or weakly positive at all coherence levels (Figure 6C). As discussed previously, this is likely a result of an increase in effort and/or attention to direction of motion signals.

Consistent with our findings in healthy controls, when the stimulus was presented passively in the non-lesioned hemisphere of patients with V1 injury (Figure 8Aii), we found that both contralesional and ipsilesional hV5/MT+ were activated and showed monotonically increasing motion-coherence dependence similar to the controls (Figure 8B). Bilateral hV5/MT+ activation with unilateral stimulus presentation in the seeing field has also been reported in other studies, such as that by Ajina et al. (2015), who also showed that the presence of the contralesioned (intact) V1 is important for promoting the communication between the two hemispheres. Performing the direction-of-motion discrimination task when the stimulus was presented in the patients’ sighted field (Figure 9A) again elicited responses commensurate to healthy subjects, suppressing coherence dependence. When the stimulus was presented passively in the blind hemifield of our patients, hV5/MT+ activation did not reach significance, in agreement with Ajina et al. (2015). However, performing the direction-of-motion discrimination task within the scotoma was sufficient to induce significant hV5/MT+ activation (Figure 10), even though subject performance remained at chance. Again, the strength of induced activity did not depend on motion coherence and is likely a result of task-related demands (Masuda et al., 2021).

On the face of it, this result may appear to be puzzling because in monkey studies (Britten et al., 1992), the response of MT neurons depends on coherence even when monkeys perform an RDK-related task. One point to consider is that electrophysiology studies measure the responses of individual units, whereas human fMRI studies measure aggregate responses of large populations of neurons with diverse properties. It is therefore possible for isolated recorded neurons to show robust coherence modulation while subjects perform a coherence-related task, while at the fMRI level, this may be masked by the activity of other units at the population level. Therefore, not identifying coherence dependence at the population level does not mean that such a dependence does not exist in select neuronal subpopulations. For example, this may happen if there are two neuronal subpopulations: one excited, the other inhibited, as a function of coherence. Moreover, if the inhibited subpopulation is more numerous than the excited one, a slight degree of inhibition per neuron may suffice, and this may be difficult to detect at a single-cell level. What we can say is that at the population level the aggregate dependence to coherence becomes weaker during task performance. A further consideration is that there are differences between the tasks used in most electrophysiology studies and our study. It is possible that performing a fine direction discrimination task, for example, as in Purushothaman and Bradley (2005), may activate different load/attentional mechanisms than simply performing a left/right direction of motion discrimination task.

### Eye Movements Do Not Explain the Effects We Observed

Although subjects were trained to fixate at the fixation mark at the center of the stimulus, the possible presence of pursuit eye movements and variable attentional shifts cannot be completely excluded. However, it is very unlikely that our results can be explained on the basis of aberrant eye movements. First, during the fixation-only task, subjects maintained high performance (>96%) on a rigorous task at fixation, suggesting they fixated well. Second, the effect of pursuit eye movements is expected to be higher at high motion coherence, yet, across subjects, responses at fully coherent RDKs were similar between the fixation-only condition (during which subjects fixated well) versus the motion discrimination condition. Furthermore, in study C, experiments were performed under eye tracking, and *post hoc* analysis confirmed that subjects maintained fixation (Supplementary Figure 5 in Papanikolaou et al., 2019). Presenting the stimuli in the sighted field of our patients shows similar results with the healthy subjects, increasing confidence that the patients were also fixating well, and the basic observations seen in healthy controls are also true for the healthy cortex of the patients.

In summary, in agreement with the literature, we found that BOLD responses in the area hV5/MT+ were monotonically increasing when subjects did not actively perform a motion discrimination task. In contrast, when subjects performed an RDK direction of motion discrimination task, hV5/MT+ BOLD responses became flat as a function of coherence, probably as a result of increased attention or task-related demands at low coherences. The same effect was observed when RDK stimuli were presented in the sighted field of the patients. When the stimulus was presented inside the patients’ scotoma, performing the motion discrimination task was necessary in order to observe significant hV5/MT+ activation. However, hV5/MT+ activation was again not stimulus coherence dependent, likely representing top-down–mediated task-dependent effects as argued by Masuda et al. (2021). Furthermore, it is reassuring that our results are consistent across basic RDK parameters and across subjects, suggesting they are not particularly sensitive to the type of lesion. In general, our observations shed further light on how visual cortex responses behave as a function of motion coherence, preparing the ground for using these methods to study visual system recovery after injury.

## Data Availability Statement

The raw data supporting the conclusions of this article will be made available by the authors, without undue reservation.

## Ethics Statement

The studies involving human participants were reviewed and approved by the IRB committees of Baylor College of Medicine and the Regierungspräsidium of the Max Planck Institute for Biological Cybernetics, Tübingen, Germany. The patients/participants provided their written informed consent to participate in this study.

## Author Contributions

AR integrated the data and together with AP wrote the final form of the manuscript. XZ and DP wrote a first draft of the manuscript. XZ, AP, and GK performed the experiments. AR, AP, and GK analyzed the fMRI data. SS, AR, and GK contributed to the conception and design of the study. AP was only involved in study C. All authors contributed to the manuscript revision, read, and approved the submitted version.

## Conflict of Interest

The authors declare that the research was conducted in the absence of any commercial or financial relationships that could be construed as a potential conflict of interest.

## Publisher’s Note

All claims expressed in this article are solely those of the authors and do not necessarily represent those of their affiliated organizations, or those of the publisher, the editors and the reviewers. Any product that may be evaluated in this article, or claim that may be made by its manufacturer, is not guaranteed or endorsed by the publisher.

## Footnotes

1
http://psychtoolbox.org

2
https://github.com/vistalab/vistadisp

3
http://surfer.nmr.mgh.harvard.edu/

4 This coherence is not related to the motion stimulus.

## Abbreviations

hV5: human Visual 5 area
MT+: middle temporal area complex
RDKs: Random dot kinematograms
fMRI: Functional magnetic resonance imaging
BOLD: blood oxygen level-dependent.
